# A Case of Cannabis-induced Acute Pancreatitis

**DOI:** 10.7759/cureus.5754

**Published:** 2019-09-25

**Authors:** Jaison John, Saurin Gandhi, Dolly Nam, Lillian Niakan

**Affiliations:** 1 Internal Medicine, Dell Medical School, The University of Texas at Austin, Austin, USA

**Keywords:** pancreatitis, cannabis, acute pancreatitis, etiological factors

## Abstract

Cannabis is one of the most commonly used illicit drugs and is now legally used recreationally or medicinally in more than half of the United States. Cannabis use has been proposed as a cause of acute pancreatitis in patients with no other identifiable etiology. Our case highlights acute pancreatitis in a young male patient with no medical problems. It is important to identify the etiology in acute pancreatitis to prevent recurrence and complications.

## Introduction

Cannabis is one of the most commonly used illicit drugs and is now legally used recreationally or medicinally in more than half of the United States [1]. Cannabis use has been proposed as a cause of acute pancreatitis in patients with no other identifiable etiology [2,3]. Recognizing the cause of pancreatitis is important to prevent recurrent episodes. We report a case of mild acute pancreatitis with no identifiable risk factors aside from heavy cannabis use in order to bring attention to the rising incidence of cannabis-induced pancreatitis.

## Case presentation

**Figure 1:**
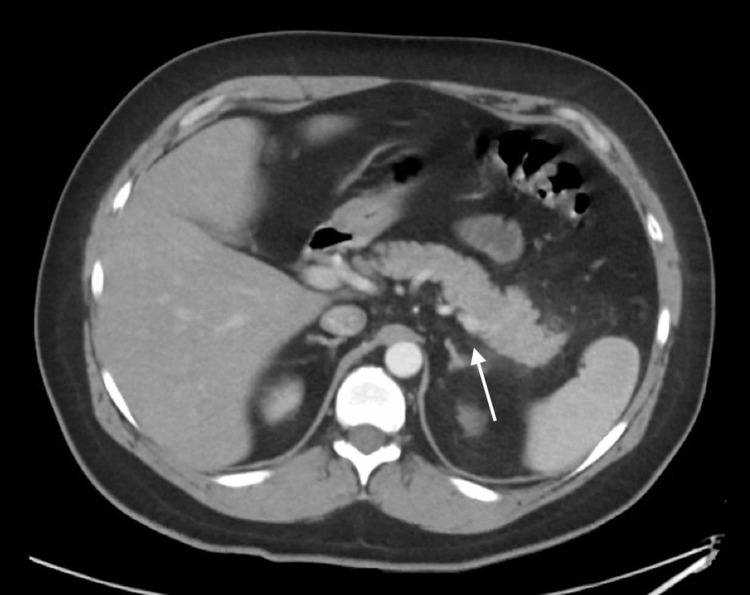
CT imaging demonstrating inflammation of the peripancreatic fat at the body and tail consistent with pancreatitis

A 41-year-old Caucasian man presented to the emergency department with a three-day history of progressively worsening constant left-sided abdominal pain. He met two of three diagnostic criteria for acute pancreatitis by Revised Atlanta Classification criteria: epigastric pain and characteristic findings on CT (lipase was normal at 36 units/L) (Figure 1). The cause for his acute pancreatitis was not clearly evident. He had no previous medical history, denied alcohol use, and quit smoking tobacco products six years ago after a prior 18-pack year history. Additionally, he denied taking any medications or having any recent trauma. Comprehensive metabolic panel was within reference range (Table 1). His triglycerides and calcium were slightly elevated at 171 mg/dL and 10.8 mg/dL, respectively. The patient’s immunoglobulin G subclass 4 (IgG4) level was normal at 26 mg/dL. Ultrasound of the abdomen did not reveal any gallstones. The patient had a 25-year history of marijuana use with increased intake over the past five years. He smokes approximately 2-3 grams of self-described high-potency marijuana daily. The calculated Naranjo adverse drug reaction probability scale score was 5 implicating marijuana as a probable cause of pancreatitis. The Naranjo adverse drug reaction probability scale was utilized to determine the likelihood of marijuana causing pancreatitis. A calculated score of 5 indicates a probable drug-induced adverse event. The patient was started on aggressive IV fluid resuscitation and had his pain managed. He had an uneventful recovery and advised to avoid further use of cannabis.

**Table 1:** Laboratory findings at the time of admission

Laboratory Test	Value (Reference)
White Blood Cell Count	20,300 mm^3^ (4,500-11,000)
Hemoglobin	15.7 g/dL (14.0-18.0)
Hematocrit	43.3% (40-54)
Platelet Count	164,000 mm^3^ (150,000-450,000)
Sodium	136 mmol/L (136-145)
Potassium	3.7 mmol/L (3.5-5.1)
Chloride	102 mmol/L (98-107)
Bicarbonate	24 mmol/L (21-31)
Blood Urea Nitrogen	10 mg/dL (6-20)
Creatinine	0.9 mg/dL (0.5-1.2)
Glucose	123 mg/dL (70-110)
Calcium	10.6 mg/dL (8.5-10.5)
Albumin	3.8 g/dL (3.2-5.5)
Total Protein	7.8 g/dL (6.7-8.2)
Alkaline Phosphatase (ALP)	94 units/L (42-121)
Aspartate Aminotransferase (AST)	17 units/L (5-34)
Alanine Aminotransferase (ALT)	23 units/L (10-60)
Total Bilirubin	1.0 mg/dL (0.2-1.2)
Cholesterol	171 mg/dL (<=200)
Triglycerides	171 mg/dL (<=150)
Lipase	36 units/L (8-78)
IgG4	26 mg/dL (1-123)

## Discussion

This case report adds to the recent growing literature that cannabis use is a possible etiology for acute pancreatitis. This is especially interesting as legalization of cannabis in many states will likely lead to increased use.

In a recent systematic review by Barkin et al. there have been 26 reported cases of cannabis-induced acute pancreatitis [2]. Recent increased cannabis use resulted in acute pancreatitis in more than half of these patients, representing a possible dose-dependent effect. Thirteen of these cases had no recurrence of pancreatitis with cessation of cannabis. Alternatively, 13 patients had recurrence with continued cannabis use.

Treatment is the same as with other causes of acute pancreatitis, however the key is identifying the underlying etiology. Having a definitive cause can prevent future episodes and reduce subsequent morbidity by avoidance of causative agents. The Naranjo adverse drug reaction probability scale is useful in determining the likelihood of a specific drug/toxin causing an adverse outcome. In our patient, we found that it was probable that cannabis indeed caused his acute pancreatitis.

Patients with idiopathic acute pancreatitis should have a thorough history taken including substance use. Identification of use of cannabis, along with length of use and dosing, can help identify the etiology of acute pancreatitis.

## Conclusions

This case report highlights a possible modifiable risk factor for acute pancreatitis, especially in younger patients when an etiology is elusive. We would recommend further studies to investigate this association given the rising use of cannabis and its legalization in many states.
